# AdmixSim: A Forward-Time Simulator for Various Complex Scenarios of Population Admixture

**DOI:** 10.3389/fgene.2020.601439

**Published:** 2020-12-03

**Authors:** Xiong Yang, Kai Yuan, Xumin Ni, Ying Zhou, Wei Guo, Shuhua Xu

**Affiliations:** ^1^Key Laboratory of Computational Biology, Chinese Academy of Sciences (CAS) and Max Planck Society (MPG) Partner Institute for Computational Biology, Shanghai Institute of Nutrition and Health, University of Chinese Academy of Sciences, Chinese Academy of Sciences, Shanghai, China; ^2^Department of Mathematics, School of Science, Beijing Jiaotong University, Beijing, China; ^3^Institute of Applied Mathematics, Academy of Mathematics and Systems Science, Chinese Academy of Sciences, Beijing, China; ^4^School of Life Science and Technology, ShanghaiTech University, Shanghai, China; ^5^Center for Excellence in Animal Evolution and Genetics, Chinese Academy of Sciences, Kunming, China; ^6^Henan Institute of Medical and Pharmaceutical Sciences, Zhengzhou University, Zhengzhou, China; ^7^Collaborative Innovation Center of Genetics and Development, Fudan University, Shanghai, China

**Keywords:** simulation, population admixture, genetic ancestry, Wright Fisher model, admixture model

## Abstract

**Background:** Population admixture is a common phenomenon in humans, animals, and plants, and it plays a very important role in shaping individual genetic architecture and population genetic diversity. Inference of population admixture, however, is very challenging and typically relies on *in silico* simulation. We are aware of the lack of a computerized tool for such a purpose. A simulator capable of generating data under various complex admixture scenarios would facilitate the study of recombination, linkage disequilibrium, ancestry tracing, and admixture dynamics in admixed populations. We described such a simulator here.

**Results:** We developed a forward-time simulator (*AdmixSim*) under the standard Wright Fisher model. It can simulate the following admixed populations: (1) multiple ancestral populations; (2) multiple waves of admixture events; (3) fluctuating population size; and (4) admixtures of fluctuating proportions. Analysis of the simulated data by *AdmixSim* showed that our simulator can quickly and accurately generate data resembling real-world values. We included in *AdmixSim* all possible parameters that would allow users to modify and simulate any kind of admixture scenario easily, so it is very flexible. *AdmixSim* records recombination break points and traces of each chromosomal segment from different ancestral populations, with which users can easily perform further analysis and comparative studies with empirical data.

**Conclusions:** *AdmixSim* facilitates the study of population admixture by providing a simulation framework with the flexible implementation of various admixture models and parameters.

## Background

Demographic history, together with natural selection, resulted in population differentiation especially in different continental populations. Nevertheless, the population admixture that has occurred over the past few millennia shaped the face of the modern world. Population isolations and migrations have been common phenomena through the history of anatomically modern humans. Hundreds of admixture events have been inferred in the recent 4,000 years in human history (Hellenthal et al., 2014), which plays an important role in shaping the genetic diversity of modern humans. The study of population admixture will shed light on the human genetic history, and has many implications in medical research. However, inferring population admixture relies heavily on simulations. *In silico* simulation is useful in testing population genetic models, studying recombination, assessing linkage disequilibrium, tracking ancestry, and evaluating admixture dynamics in admixed populations. Many forward- and backward-time simulators (Hudson, 2002; Guillaume and Rougemont, 2006; Liang et al., 2007; Hernandez, 2008; Chen et al., 2009; Hoban et al., 2011) have been developed in recent years. Some backward-time simulators (most of which are coalescent-based), for example, *ms* (Hudson, 2002), can simulate population admixtures in simple scenarios. However, it is very difficult or even impossible to simulate an admixed population with a fluctuating population size or fluctuating gene flow generation after generation. Some forward-time simulators, for example, *SFS_CODE* (Hernandez, 2008), can also simulate admixtures in simple scenarios, but they suffer from the same problems as coalescent-based simulators. To our knowledge, there is no simulator that focuses on simulating and tracking the dynamics of recombination and ancestry in admixed populations, and allowing change population size and gene flow generation after generation.

## Implementation

### A Generalized Admixture Model

Population admixture occurs when gene flow moves from ancestral populations either continuously or discontinuously. To render the modeling of population admixture more general, we can model this process generation by generation, in which, if the admixed population does not receive further gene influx in a particular generation, we set the strength of gene flow to 0. A given admixed population with *K* (*K* > 1) ancestral populations formed *T* (*T* > 0) generations ago can be fully modeled by a *K* × *T* matrix *M*. The rows (*i*) refers to the ancestral populations and the columns (*j*) refers to the generations. *m*_*ij*_ in *M* denotes the strength of gene flow from the *jth* ancestral population at *ith* generation, where *m*_*ij*_ fulfills two requirements: (1) 0 ≤ *m*_*ij*_ ≤ 1; and (2) ∑*m*_*ij*_ = 1 when *i* = 1.

### Simulation Process

Because novel mutations and selections have negligible impacts on shaping the genetic diversity of a recent admixed population on the whole genome scale, we disregarded mutations and selections in our simulation here. Recombination is modeled as a Poison process along the chromosome (chromosomal end is ignored) with rate 1 (unit in Morgan). At a specific generation, *i*, the size of the admixed population is *N*, and the rate of gene flow from the *jth* ancestral population is *m*_*ij*_, we generate individuals in the current generation by two steps.

1. For gene flow from the *jth* ancestral population, we randomly sample *N*×*m*_*ij*_ individuals from the *jth* ancestral population and repeat this procedure for all the gene flow events in the current generation, and the rest of the individuals are randomly sampled from the admixed population in the previous generation;
2. With the sample pool generated in step 1, we randomly choose two individuals in the pool, then randomly choose one chromosome from one individual. We pair and recombine it with the one randomly chosen in another individual to form a new chromosome pair. We repeat these procedures until *N* individuals are generated.

Individuals are generated using the two steps generation by generation. At the end of the simulation, *n* individuals are randomly sampled; and the start and end positions and the ancestry of each chromosome segment are recorded. The haplotypes for each individual are also recorded for further study.

## Results

**Figure 1:**
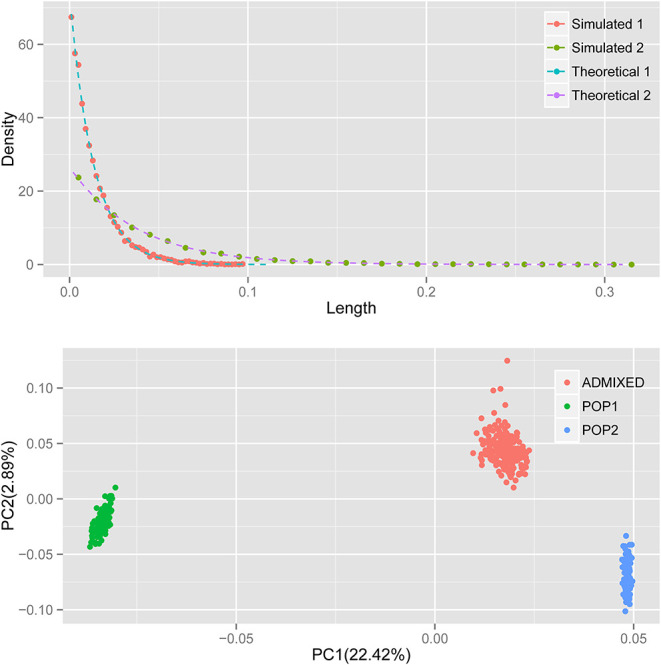
The distribution of LACS and PCA of the simulated data. Top: red and green dots denote the distribution of LACS from ancestral population 1 and ancestral population 2; blue and purple dashed lines are the theoretical distributions of LACS derived from *(1-m)Te*^−(1−*m*)*Tx*^, where *m* is the ancestry contribution of that ancestral population and *T* is the generation since admixture. Bottom: green and blue dots denote the individuals from ancestral populations, and the red dots denote individuals in the admixed population.

A previous study established the theoretical distribution of the length of the ancestral chromosomal segment (LACS) under an HI model (Jin et al., 2014). It is not difficult to test the performance of our simulator. Under the HI model, the distribution of LACS from the ancestral population with ancestry contribution *m* is *(1-m)Te*^−(1−*m*)*Tx*^. Here, we simulated an admixed population with constant *N* = 5,000, admixed 100 generations ago, following the HI model, in which ancestral population 1 contributes 25% of the total ancestry. As expected, the distribution of LACS simulated (dots) matches the theoretical distribution of LACS (dashed line) well for ancestries from both ancestral populations 1 and 2 (Figure 1).

Principal component analysis (PCA) with *smartpca* (Patterson et al., 2006; Price et al., 2006) shows that first principal component (PC) separates ancestral population 1 and ancestral population 2, just as expected and usually observed in real data. Simulated individuals from the two ancestral populations cluster within their own groups, and the admixed individuals cluster between two ancestral populations along PC1 (Figure 1). Because ancestral population 2 contributes more to the admixed population, the distance between individual from admixed population and individual from ancestral population 2 is much closer than that from ancestral population 1 to the admixed individuals. All these results observed in the simulated data resembled what can be observed in real data, which indicates our simulator does generate correct datasets that resemble the real one.

In the results of the *ADMIXTURE* analysis (Alexander et al., 2009) of the simulated data, we can clearly observe the assignment of ancestries of the admixed individuals: blue represents ancestry from ancestral population 1 and green represents ancestry from ancestral population 2. The contributions from ancestral population 1 were found to vary from 18 to 30% (Supplementary Figure 1), which also resembles what was observed in real data.

**Figure 2:**
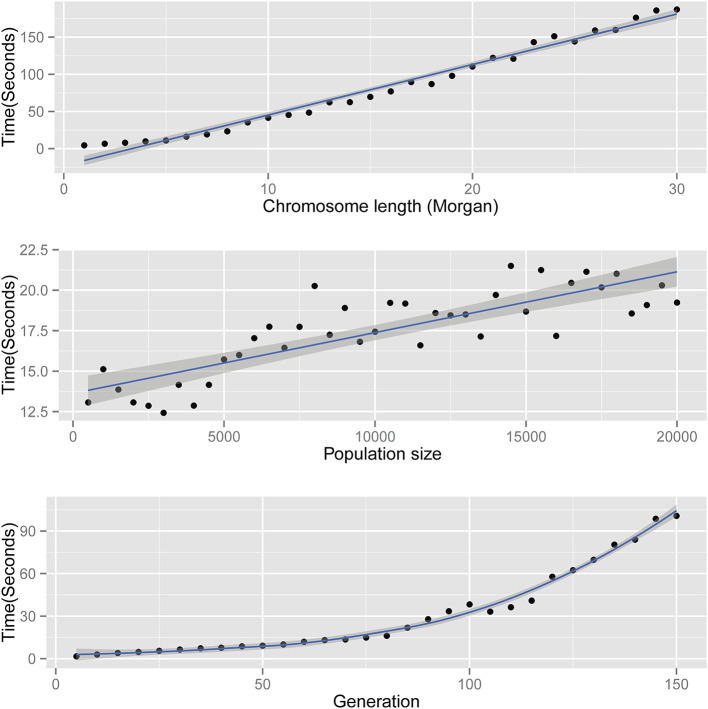
Running time of *AdmixSim*. Top: running time versus chromosome length simulated; middle: running time vs. population size simulated; and bottom: running time vs. generation since admixture.

The time complexity of *AdmixSim* is *O(L), O(N)*, and *O(T*^2^*)*. In theory, the running time of *AdmixSim* is linearly determined by the chromosome length (*L*) and the population size (*N*) simulated; and quadratic depends on the generations since admixture (*T*). Benchmark tests are carried on a laptop with an *Intel Core*^*TM*^ *Duo* CPU @ 2.0 GHz, 2 Gb RAM, and Ubuntu 12.04 32-bit operation system. Running time is recorded by Linux command *time*, and the *user* time is collected and compared. Each time, only one parameter is allowed to be variable. For example, to assess the impact of generation since admixture on running time, we ran a series of tests in which generation ranges from start to end, increased by 1 step size each time. The details of the parameter settings for each test can be found in Table 1. The results show that the *AdmixSim* runs very fast even with large population size, and the running time shows a trend as expected (Figure 2).

**Table 1:** Detailed parameters of benchmark test.

**Parameter**	**Start**	**End**	**Step size**	**Other fixed settings**
Generation (*T*)	5	150	5	*N* = 5,000, *K* = 2, *L* = 1, and *n* = 200
Chromosome length (*L*)	1	30	1	*N* = 5,000, *K* = 2, *T* = 20, and *n* = 200
Population size (*N*)	500	20,000	5	*K* = 2, *L* = 1, *T* = 20, and *n* = 200

*First column is the parameter to be tested; the second and the following two columns are the ranges of the parameters (start, end, and increasing step); last column is all other parameters setting to constants. N is population size, T is generation since admixture, K is the number of ancestral populations, L is the chromosome length (unit in Morgan), and n is the number of individuals sampled from admixed population at the end of simulation*.

## Conclusions

Here, we developed a fast and flexible simulator suitable for modeling various and complex scenarios involving population admixture. This system can simulate an admixed population under several sets of conditions: (1) multiple ancestral populations; (2) multiple waves of admixture events; (3) fluctuating population sizes; and (4) fluctuating admixture proportions. With the *AdmixSim*, the user can not only easily simulate an admixed population under the three typical admixture models, i.e., hybrid isolation (HI) model, gradual admixture (GA) model, and continuous gene flow (CGF) model as described in previous studies (Jin et al., 2012), but also simulate admixed populations with more complex scenarios, for example, three-way admixture with multiple waves of gene flow. In several recent studies, we have applied this simulator to generate simulation data under many of population admixture scenarios, such as GA model, CGF model, and multiple-wave admixture model (Ni et al., 2016, 2018, 2019; Feng et al., 2017). The distribution of ancestral segments based on simulated data matched well with the theoretical distribution, which showed the reliability and power of our simulator. This simulator will facilitate the study of population admixture and greatly help us to understand the processes of human migration, admixture, and evolution, which provides further insight into both evolutionary and medical studies of human genetic diversity.

## Data Availability Statement

The original contributions presented in the study are included in the article/Supplementary Material, further inquiries can be directed to the corresponding author/s.

## Author Contributions

SX conceived the study. XY designed and implemented the *AdmixSim* with contribution from XN, WG, and YZ. XY and SX wrote the manuscript. XY, XN, and WG analyzed the time complexity and the performance of the simulator with contribution from KY. All authors read and approved the final manuscript.

## Conflict of Interest

The authors declare that the research was conducted in the absence of any commercial or financial relationships that could be construed as a potential conflict of interest.

## Supplementary Material

Supplementary Figure 1 *ADMIXTURE* analysis of simulated data. Blue and green denote two ancestral components and the admixed individuals show combination of the ancestries from two ancestral populations.

Supplementary Text 1 Tutorials for *AdmixSim*.

## Abbreviations

CGF: Continuous gene flow
GA: Gradual admixture
HI: Hybrid isolation
LACS: Length of ancestral chromosomal segment
PCA: Principal component analysis
PC: Principal component.
