# Supplementary figures and images for: AdmixSim: A Forward-Time Simulator for Various Complex Scenarios of Population Admixture
Source: Front Genet. 2020 Dec 3;11:601439. doi: 10.3389/fgene.2020.601439 (PMC7744625; doi:10.3389/fgene.2020.601439)

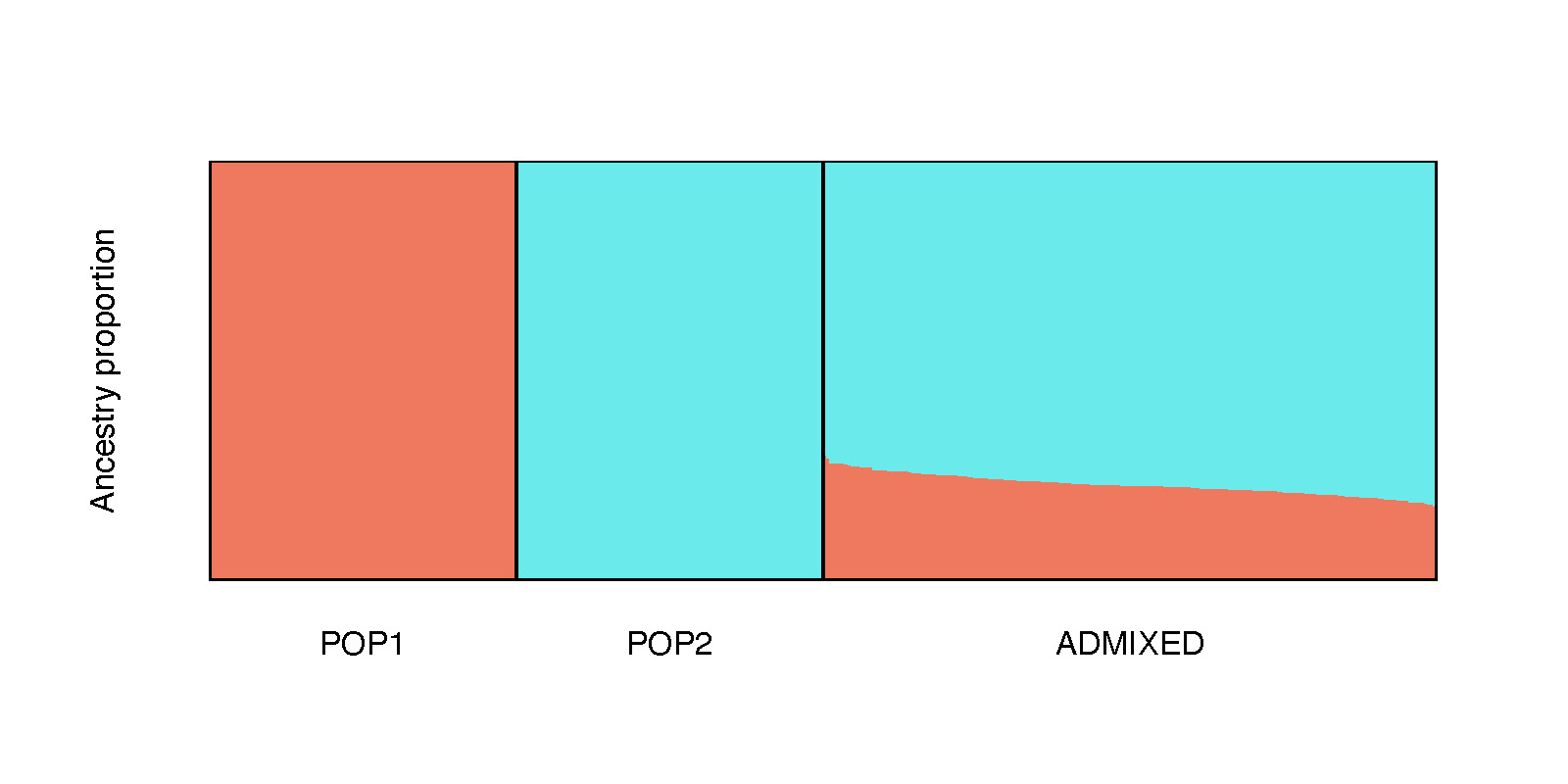

Supplement: Supplementary Figure 1 — ADMIXTURE analysis of simulated data. Blue and green denote two ancestral components and the admixed individuals show combination of the ancestries from two ancestral populations. [file Image_1.JPEG]
