# Supplementary material for: AdmixSim: A Forward-Time Simulator for Various Complex Scenarios of Population Admixture
Source: Front Genet. 2020 Dec 3;11:601439. doi: 10.3389/fgene.2020.601439 (PMC7744625; doi:10.3389/fgene.2020.601439)
Supplement: Supplementary Text 1 — Tutorials for AdmixSim. [file Table_1.DOCX]

**Tutorial for AdmixSim**

**Descriptions**

1. Population model assumptions

The population in each generation evolves following standard Wright Fisher model without mutation and selection. That is, randomly sample two individuals from the population, and randomly choose one of the chromosomes in each individual, pair them and recombine to form a new chromosome pair for next generation. Repeat this process until sampled *N* chromosome pair. Here *N* denotes population size in specific generation.

Recombination is modeled as a Poisson Process along the chromosome with rate 1 (Unit in Morgan).

1. AdmixSim description

Here implemented a very flexible simulator for admixed population, in which:

1) Can take arbitrary number of ancestral populations;

2) Can take arbitrary waves of population admixture;

3) Population size can be changed generation by generation;

4) And admixture proportions can also be changed generation by generation.

**Get started**

1. Requirements

To run the simulator, java 1.6 or upper is required.

1. Get command line help

*java -jar AdmixSim.jar –h* or *java -jar AdmixSim.jar --help*

1. Run it with the toy data

*java -jar AdmixSim.jar --gen 10 --nanc 2 --len 1.0 --file toy.par --samp 20 --input test --output test-out*

Explanation: The toy command simulate an admixed population starts to admix 10 generation ago (*--gen 10*), with two ancestral populations (*--nanc 2*), the chromosome length simulated is 1.0 (*--len 1.0*), the detailed model description is in file toy.par (*--file toy.par*), at the end of simulation, sample 20 individuals from the admixed population (-*-samp 20*), the prefix of input files (*test.hap* and *test.map*) is test (-*-input test*) and the prefix of output files is test-out (*--output test-out*). Details of each file will explain in next section.

**Input and output**

1. Input files

1). Model description file

Firstly, in model description file set up initial number of haplotypes to be sampled from the ancestral populations

Secondly, set up the population size and ancestral proportions for each generation in one line

Note: anything follows "#" is treated as comment

Here is a complete example:

#set up number of haplotypes in each ancestral population to be sampled from

10 10

// #indicates start of population size and admixture proportions

100 0.5 0.5 #init population, two ancestral population, each contribute 50%

100 0 0

100 0.1 0 #second wave, with 10% extra gene flow from ancestral population 1

100 0 0

100 0 0

100 0 0.2 #third wave, with 20% gene flow from ancestral population 2

100 0 0

150 0 0 #population increase to 150

100 0 0 #population size decrease to 100

100 0 0

By the same manner, it's quite simple to implemented HI, GA or CGFR or CGFD models as described in *Jin Wenfei et al (2012) AJHG*.

HI model:

10 10

//

100 0.7 0.3 #init population, two ancestral population, contribute 70% and 30%

100 0 0

......

100 0 0

GA model:

10 10

//

100 0.7 0.3 #init population, two ancestral population, contribute 70% and 30%

100 0.1 0.1

......

100 0.1 0.1

CGFR model:

10 10

//

100 0.7 0.3 #init population, two ancestral population, contribute 70% and 30%

100 0.1 0

......

100 0.1 0

CGFD model:

10 10

//

100 0.7 0.3 #init population, two ancestral population, contribute 70% and 30%

100 0 0.1

......

100 0 0.1

2). Map file

The genetic positions for each marker are given in Morgan, one line per marker.

Here is an example:

0.00097100

0.00238066

0.00367538

......

3). Haplotype file

The haplotypes of ancestral populations to be sampled from are combined in one file, one haplotype per line. The first *n1* lines correspond to haplotypes for first ancestral population, second *n2* lines correspond to haplotypes for second ancestral population and so on. In which the number of ancestral populations and the numbers of haplotypes for each ancestral population ((*n1, n2* and so on) are given in model description file.

Here is an example:

10110000001001000010000000101101000101010000110101110000000000100000

00100000100001000010000000101101010100110000010111110010000000100000

00100000100001000010000000101101010100010100010101110000001000110000

00100000100001000010100000101101010100010000010101110000001000100000

......

01000000000001101000000100000001000100000010000000010100010000100001

Hints: the map file and haplotype file should have the same prefix, for example*, test.map* and *test.hap*

1. Output files

1). Admixed haplotypes

Record the haplotypes from the individuals sampled from the admixed population, format is the same as input haplotype file

2). Segment file

In which record the start, end and which ancestral that segment comes from. Each line corresponds to one chromosomal segment.

Here is an example:

0.00000000 0.07785695 2

0.07785695 0.30178126 1

......

0.30178126 0.41594482 2

**Complete arguments list**

-h/--help print help message [optional]

-f/--file model description file [required]

-i/--input prefix of input files [required]

-g/--gen generations since admixture [optional, default: 1]

-k/--nanc number of ancestral populations [optional, default: 2]

-l/--leng length of chromosome to be simulated [optional, default: 1.0]

-n/--samp number of individual(s) to be sampled [optional, default: 10]

-o/--output prefix of output files [optional, default: output]

-s/--seed seed of random generator [optional, default: current time]

**Easily couple with other simulator**

It's very easy to couple with other simulator such as *ms*. For example, using *ms* to simulate two ancestral populations, whose Ne remains constant, i.e. Ne=5000, split 4000 generations ago, command as below:

*ms 200 1 -t 2000 -r 2000 10000000 -I 2 100 100 -ej 0.2 2 1 -p 8 > SimAnc.txt*

Then just simply convert into the files needed in our simulator:

*python convert.py simAnc.txt simAnc 200*

It will produce the map file and the ancestral haplotype file:

simAnc.map simAnc.hap

Afterwards, the simulated ancestral haplotypes could be used in our simulator, for example:

*java -jar AdmixSim.jar -g 20 -k 2 -l 2 -f sim1.par -n 100 -i simAnc -o sim1*
